# Waste-Glass-Derived Porous Silica: Synthesis and Structural Characterization

**DOI:** 10.3390/ma19030601

**Published:** 2026-02-04

**Authors:** Antônia Millena de Oliveira Lima, Manuel Pedro Fernandes Graça, Imen Hammami, Ana Angélica Mathias Macêdo

**Affiliations:** 1 Instituto Federal de Educação Ciência e Tecnologia do Maranhão, Campus Imperatriz, São Luís 65919-050, Brazil; amillenalima@hotmail.com (A.M.d.O.L.); anaangellica@yahoo.com.br (A.A.M.M.); 2 Foundation for Research and Scientific and Technological Development of Maranhão (FAPEMA), São Luís 65075-340, Brazil; 3 i3N and Physics Department, University of Aveiro, 3810-193 Aveiro, Portugal

**Keywords:** waste glass, sodium hydroxide, silica gel, mesoporous

## Abstract

This work reports the reuse of waste glass as a sustainable silica source for the synthesis of mesoporous silica, as an alternative to conventional silica precursors. Silica gel was produced through alkaline dissolution of glass powder using sodium hydroxide and subsequently employed as a precursor for the synthesis of porous silica. The waste-derived glass powder and the synthesized silica-based materials were characterized to assess their structural, morphological, surface, and textural properties. XRD analysis confirmed the amorphous nature of all samples, while FTIR spectra indicated successful silica network formation with modifications in bond connectivity. SEM imaging revealed spherical particles with average diameters of approximately 0.19 ± 0.02 µm for silica gel and 0.15 ± 0.03 µm for the mesoporous silica. Zeta potential measurements indicated a negative surface charge and good colloidal stability in aqueous media. Nitrogen sorption analysis revealed that the specific surface area was limited by the low solubility of silica gel in acidic media, which prevents ideal condensation on the surface of surfactant micelles. The results demonstrate that waste glass-derived silica gel is a promising precursor, although the synthesis conditions did not yield a highly ordered mesostructure, highlighting the need for further control of precursor solubility and pH.

## 1. Introduction

Soda–lime–silica glass, commonly referred to as soda–lime glass, is the most prevalent type of glass found in urban solid waste. Its structure is primarily composed of tetrahedral SiO_4_ units, with a typical composition of approximately of 73% SiO_2_, 15% Na_2_O, 7% CaO, 4% MgO, and 1% Al_2_O_3_ (wt%) [1]. The high silica (SiO_2_) content in these compositions favors the reuse of soda–lime glass as a silicon source in the synthesis of amorphous silica. Liu et al. (2019) compiled several compositions for discarded glass and identified their main sources, highlighting glass bottles as one of the largest contributors to glass waste streams [2].

This dominance of bottle-derived soda–lime glass has been further intensified by recent changes in consumption patterns. During the COVID-19 pandemic, beer consumption shifted predominantly toward disposable bottles as domestic consumption replaced social gatherings in bars. Reports indicate that approximately 70% of beer sales in Brazil relied on disposable bottles, in contrast to pre-pandemic trends. This shift led to an increase in demand for glass production in Brazil, as the beer industry traditionally relied on returnable bottle logistics. Consequently, a shortage of glass emerged, affecting large industrial sectors, particularly the beverage industry [3,4].

Given that a large fraction of these bottles are non-reusable and end up discarded, there is an urgent socio-environmental need to develop high-value upcycling strategies. A promising solution lies in transforming the discarded soda–lime glass into silica precursors for the synthesis of silica-based materials. Conventionally, the synthesis of high-order mesoporous silica relies on alkoxide precursors such as tetraethyl orthosilicate (TEOS) and tetramethyl orthosilicate (TMOS) [5,6]. However, the production of these traditional precursors is characterized by high energy consumption, high costs, and the use of toxic organic solvents, which results in a significant environmental footprint. Consequently, there is growing interest in cost-effective, biocompatible, and eco-friendly alternatives, such as waste-derived soda–lime glass, as sustainable silicon sources [7].

Mesoporous silica consists of a three-dimensional network of SiO_4_ tetrahedra linked by siloxane bonds, forming an inorganic polymeric structure with well-defined pore sizes in the mesoporous range (2–50 nm), where variations in network connectivity and the presence of modifier species strongly influence the structural, dielectric, and transport properties of silicate-based amorphous materials [8]. The synthesis of such materials typically involves the use of structure-directing agents, such as surfactants, which template the formation of pores. After removal of the surfactant, the silica surface is predominantly covered by silanol (Si–OH) groups, although residual siloxane groups may remain. The surface properties of silica are governed by the density and distribution of these functional groups and can be further tailored through surface modification or functionalization, enabling the design of materials with application-specific properties [9,10,11,12,13]. Previous studies have demonstrated that glass-waste streams can be effectively converted into soluble silicate precursors to produce high-value porous and mesoporous silicas. For example, recycled glass cullet has been upcycled into highly porous silica microspheres (porosity ≈ 69%), which showed excellent performance in the adsorption of organic dyes for wastewater treatment [14]. In addition, waste-derived siliceous materials have been reported to yield high-purity amorphous silica with specific surface areas of several hundred m^2^/g [15,16], exhibiting catalytic and adsorption performances comparable to those obtained using commercial silicon precursors. More recently, glass waste has been successfully employed as a silicon source to obtain high-purity silica (up to 99.4%) for the synthesis of 45S5 bioglass intended for biomedical applications [17]. These results highlight the versatility and effectiveness of glass-waste recycling strategies in both environmental remediation and biomedical engineering.

Among the various types of mesoporous silicas, SBA-15 (Santa Barbara Amorphous-15) is one of the most extensively studied structures due to its ordered two-dimensional hexagonal pore arrangement, relatively large pore diameters, and thick pore walls that confer enhanced mechanical and hydrothermal stability [18,19]. Typically synthesized using the triblock copolymer Pluronic P123 as a template in acidic media, SBA-15 features large mesopores (5–30 nm) and a complementary micropore network [20,21]. These characteristics make it exceptionally suitable for the immobilization of large biomolecules and drug delivery systems [22,23]. However, the high cost of traditional silicon sources for SBA-15 synthesis remains a barrier to large-scale application, further justifying the investigation of waste-derived alternatives.

In this context, the present work investigates the reuse of “long-neck” glass bottles to produce silicon precursors for the synthesis of SBA-15-type mesoporous silica, serving as a sustainable alternative to traditional precursors. Long-neck glass bottles are typically manufactured from soda–lime–silica glass, whose composition is broadly consistent across beverage containers. According to literature, these glasses generally contain 70–74 wt% SiO_2_ as the network former, 12–15 wt% Na_2_O as the main flux, 9–12 wt% CaO as the principal stabilizer, and small amounts of MgO and Al_2_O_3_ [24]. Transition-metal oxides, such as iron or chromium, may be present in green or amber bottles to provide coloration and light protection. Several characterization techniques were used to evaluate the effectiveness of these waste-derived precursors. X-ray diffraction (XRD) was used to confirm the preservation of the amorphous state, while Fourier transform infrared spectroscopy (FTIR) investigated the evolution of Si–O–Si and Si–OH bonding environments. Zeta potential measurements assessed the surface charge and colloidal stability, which are critical for dispersion behavior in biomedical applications. Finally, nitrogen (N_2_) adsorption analysis was used to evaluate the textural properties and the impact of silica gel solubility on the development of the mesoporous framework. While challenges regarding solubility in acidic media were identified, this method represents a significant step toward the sustainable valorization of post-consumer glass waste.

## 2. Materials and Methods

### 2.1. Preparation of Silica Gel and SBA-15-Type Mesoporous Silica from Waste Glass

**Figure 1:**
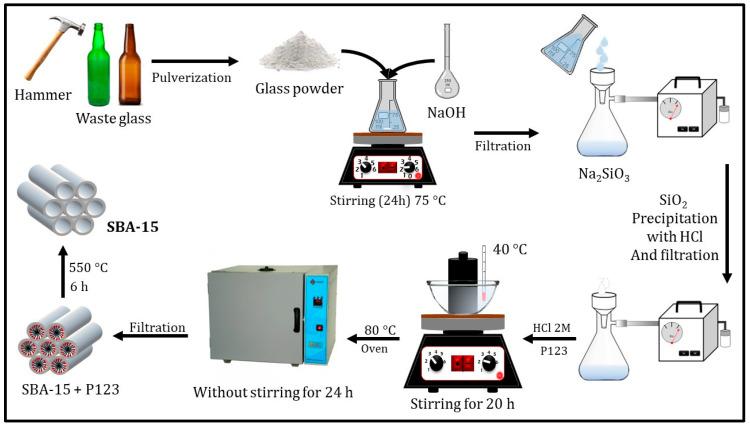
Schematic representation of the synthesis route used to convert post-consumer waste glass into silica gel and mesoporous SBA-15-type mesoporous silica, including glass pulverization, alkaline dissolution in NaOH, silica gel precipitation by acidification, and subsequent hydrothermal synthesis and calcination.

Glass bottles were collected from local businesses in the city of Imperatriz (MA, Brazil). The bottles were initially cleaned with running water and detergent to remove surface contaminants and adhesive residues, followed by rinsing with distilled water and drying at room temperature. The dried bottles were manually crushed using a hammer and subsequently pulverized with a mortar and pestle to obtain a fine glass powder. While this approach is suitable for laboratory-scale preparation, ball milling represents a scalable and industrially relevant alternative for producing glass powder in larger quantities. This method is easily replaced by using a ball mill for large-scale production. The experimental procedure for converting these waste glass precursors into silica gel and mesoporous SBA-15 is schematically illustrated in Figure 1.

The amount of 5 g of glass powder was added to 30 mL of an aqueous sodium hydroxide solution (6 mol·L^−1^ NaOH), promoting the alkaline dissolution of silica according to reaction (1):

SiO_2_(s) + 2NaOH(aq) ⟶ Na_2_SiO_3_(aq) + H_2_O(l) (1)

The suspension was maintained under constant stirring at 75 °C for 24 h. Following the reaction, the mixture was filtered to obtain a sodium silicate solution. Silica gel was then precipitated by adding concentrated hydrochloric acid (HCl). The resulting white silica gel was aged overnight at room temperature. As summarized in Figure 1, the obtained silica gel was subsequently used as the silicon source for SBA-15-type mesoporous silica preparation.

Mesoporous silica SBA-15 was synthesized using a hydrothermal method adapted from Rechotnek et al. [25]. Briefly, 4 g of the template Pluronic P123 (CAS No. 9003-11-6; EO_20_PO_70_EO_20_) were dissolved in a mixture of 20 mL of HCl and 130 mL of distilled water under stirring for 1 h. The temperature of the solution was then increased to 45 °C. Subsequently, 8.5 g of the waste-derived silica gel was added to the reaction medium, and the mixture was stirred for 20 h. The resulting suspension was then kept in a preheated oven at 80 °C for 24 h under static conditions. The solid product was collected by vacuum filtration, washed with 1 L of distilled water, and dried at 55 °C for 12 h. Finally, the white solid was calcined at 550 °C for 6 h to remove the organic template. The resulting mesoporous material was designated as MS-GP, corresponding to a P123-templated SBA-15-type mesoporous silica derived from waste glass, without long-range hexagonal ordering.

The materials synthesized and characterized in this study, along with their respective identification codes, are summarized in Table 1. In this study, the term SBA-15-type mesoporous silica refers to materials synthesized using the conventional P123-templated acidic route, although long-range hexagonal ordering was not achieved.

### 2.2. Characterization Methods

X-ray diffraction (XRD) patterns were recorded using a Rigaku Miniflex II diffractometer (Rigaku Corporation, Tokyo, Japan) equipped with Cu Kα radiation (λ = 1.5406 Å). Data were collected in the 2θ range of 10–80° with a step size of 0.02° and a counting time of 2 s per step.

Fourier-transform infrared (FTIR) spectra were recorded in transmission mode in the range of 400–4000 cm^−1^ using an Alpha II FTIR spectrometer (Bruker Optics, Ettlingen, Germany). Samples were prepared as KBr pellets by mixing the powder with KBr at a mass ratio of 1:200 mg.

The surface morphology of the prepared samples was investigated by scanning electron microscopy (SEM) using a TESCAN Vega 3 microscope (TESCAN, Brno, Czech Republic). Before imaging, the samples were coated with a thin layer of gold (around 10 nm) using a sputter coater to ensure surface conductivity and prevent charging during analysis. The elemental composition of the samples was analyzed semi-quantitatively by energy-dispersive X-ray spectroscopy (EDS) using a Bruker QUANTAX EDS (Bruker Nano GmbH, Berlin, Germany) system coupled to the SEM, operated at an accelerating voltage of 20 kV. Particle size distribution was determined from SEM images by measuring the diameters of particles using ImageJ software 1.8.0.

Zeta potential (ζ) measurements were performed using a Malvern Zetasizer Nano ZL instrument (Malvern Panalytical Ltd., Malvern, UK). For each measurement, 2.0 mg of sample was suspended in 10.0 mL of phosphate-buffered saline (PBS) at pH 7.4 and sonicated for 5 min to ensure homogeneous dispersion before analysis.

Surface area and pore size distributions were determined using N_2_ adsorption–desorption isotherms obtained at −196 °C on a Micromeritics Tristar II 3020 system (Micromeritics Instrument Corp., Norcross, GA, USA). The specific surface area was calculated using the Brunauer–Emmett–Teller (BET) method [26], while the pore size distribution and volume were derived from the adsorption branch of the isotherms using the Barrett–Joyner–Halenda (BJH) method [27].

## 3. Results and Discussion

### 3.1. SEM and EDS Characterization

**Figure 2:**
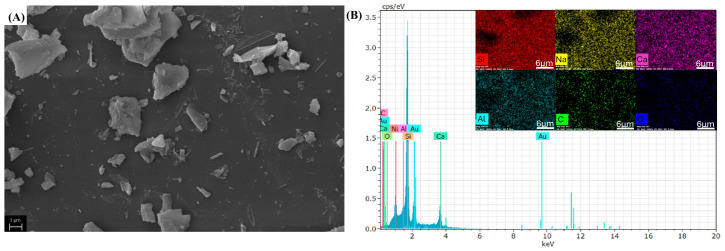
(**A**) SEM images and (**B**) EDS spectra and elemental mapping for the glass powder (GP) sample.

The surface morphology and elemental composition of the samples were evaluated using SEM and EDS analysis (Figure 2).

As shown in Figure 2A, the glass powder (GP) exhibits an inhomogeneous morphology composed of angular and irregular flaky particles. This irregular morphology is typical for mechanically crushed waste glass, as previously reported in the literature [28]. EDS analysis, presented in Figure 2B, confirms the presence of Si, O, Na, Ca, and Al, consistent with the typical composition of soda–lime–silica glass.

**Figure 3:**
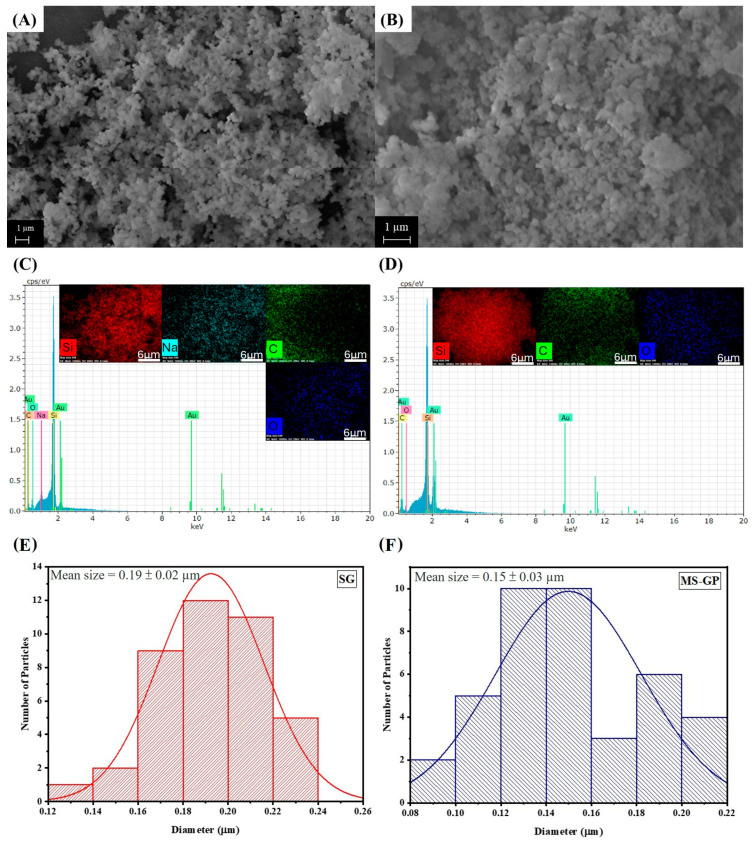
SEM images, EDS mapping, and particle size distributions for (**A**,**C**,**E**) SG and (**B**,**D**,**F**) MS-GP samples.

The silica gel (SG), obtained after alkaline dissolution and acid precipitation, shows a significant morphological transformation (Figure 3A). The SG sample consists of well-distributed, spherical particles with a mean diameter of approximately 0.19 ± 0.02 µm, as determined from the particle size distribution (Figure 3E). This morphology is commonly observed for silica gels prepared using sol–gel routes [29,30,31]. The EDS results, illustrated in Figure 3D, indicate that while the Al and Ca present in the GP precursors were successfully removed during the purification process, Na atoms persist within the structure despite the HCl precipitation.

For MS-GP (Figure 3B), a spheric morphology similar to that of the SG sample was observed, with a slightly reduced mean particle size of 0.15 µm ± 0.03 (Figure 3F). Although SBA-15 materials are reported to exhibit a rod-like structure [32], the retention of a spherical form in this study is attributed to the pH conditions maintained during the synthesis involving the P123 template. It is noteworthy that sphere-like mesoporous silicas are highly desirable for applications in high-performance liquid chromatography (HPLC) as this geometry facilitates uniform column packing and enhances the separation efficiency of diverse molecular species [33,34]. The EDS mapping and spectra for MS-GP (Figure 3D) show a high-purity framework composed predominantly of Si and O atoms. The absence of Na in the final MS-GP structure indicates that the residual sodium ions found in the SG sample were effectively leached out during the hydrothermal treatment and final washing steps of the SBA-15-type mesoporous silica synthesis. Additionally, the Au peaks observed in all EDS spectra are attributed to the gold-sputtering process required for sample conductivity during analysis.

### 3.2. XRD Analysis

**Figure 4:**
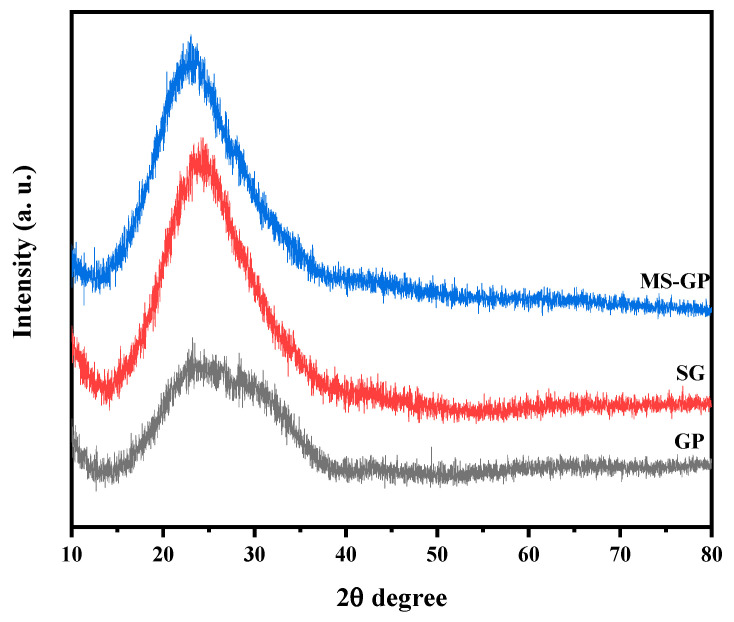
XRD patterns for GP, SG, and MS-GP samples.

Figure 4 presents the XRD patterns of the GP, SG, and MS-GP samples. All samples exhibit a characteristically amorphous structure, evidenced by the absence of sharp diffraction peaks. Each pattern displays a prominent broad hump centered at approximately 2θ = 25°, which is attributed to the short-range structural order within the disordered silica network [13]. This observed behavior is consistent with the literature for silica-based materials, confirming that the glassy state was maintained throughout the chemical transformation from glass powder to silica gel and mesoporous materials [35,36].

**Figure 5:**
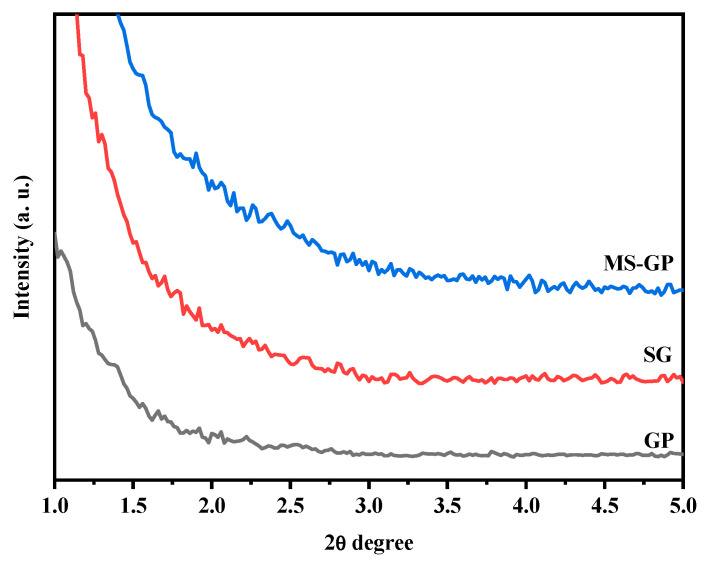
Small-angle XRD patterns for GP, SG, and MS-GP samples.

To evaluate the structural ordering of the mesoporous framework, small-angle XRD patterns were recorded and are shown in Figure 5. In general, highly ordered mesoporous silicas, such as SBA-15, exhibit three well-resolved peaks at low angles, corresponding to the (100), (110), and (200) reflection planes of a 2D hexagonal (p6 mm) symmetry [37,38]. However, these characteristic reflections are absent in all analyzed samples. While the lack of ordered reflections was expected for the GP and SG samples, the absence of these peaks in the MS-GP sample suggests a disordered mesostructure. This result can be attributed to the limited solubility of the waste-derived silica gel in the acidic reaction medium during the synthesis. This incomplete dissolution hindered the effective condensation and organization of silica species around the surfactant micelles, preventing the formation of a highly periodic hexagonal arrangement.

### 3.3. FTIR Analysis

**Figure 6:**
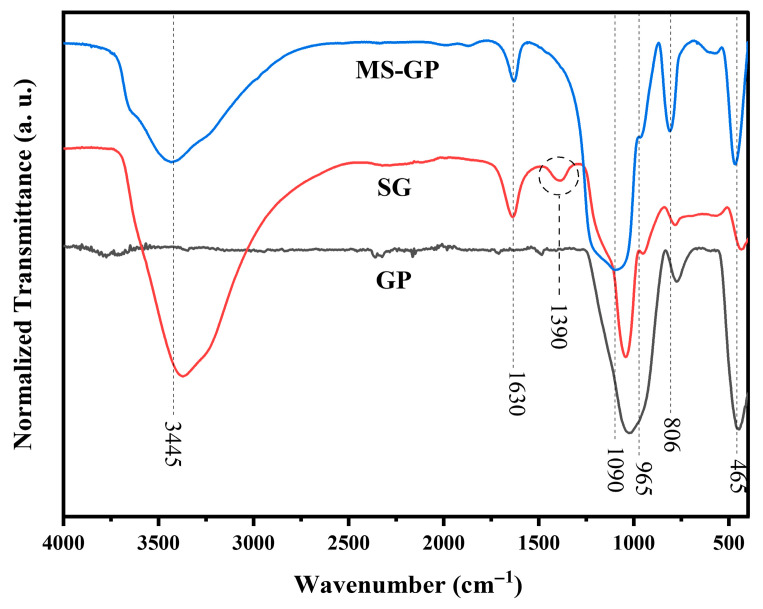
FTIR spectra for GP, SG, and MS-GP samples.

Figure 6 presents the FTIR spectra of the GP, SG, and MS-GP samples. All spectra exhibit characteristic bands associated with the silica network. The bands centered at approximately 1090 cm^−1^, 806 cm^−1^, and 465 cm^−1^ are attributed to the asymmetric stretching, symmetric stretching, and bending vibrations of Si–O–Si bonds in siloxane groups, respectively [39]. The band located at around 965 cm^−1^ is assigned to the stretching vibration of Si–OH groups. In addition, the broad band at around 3445 cm^−1^ and the band at 1630 cm^−1^ correspond to the stretching and bending vibrations of O–H bonds, arising from both surface silanol groups and adsorbed water molecules [40,41].

Differences between the GP and SG spectra confirm the formation of silica gel following alkaline dissolution and acid precipitation. The increased absorbance of the band at around 3400 cm^−1^ in the SG sample indicates a higher concentration of silanol groups within its structure. Additionally, the SG sample exhibits a distinct band at 1390 cm^−1^, which can be attributed to residual carbonates (CO_3_^2−^) resulting from the interaction of the alkaline silicate solution with atmospheric CO_2_ during processing and drying [42,43].

Significant shifts observed in the siloxane-related bands (1090, 806, and 465 cm^−1^) for the SG sample compared to the GP precursor are attributed to the structural reorganization of the silicate network during the sol–gel transition. While EDS identified Al in the GP sample (Figure 2B), its absence in the SG sample (Figure 3D) indicates a successful chemical purification during the acid precipitation and washing steps, where soluble Al species were removed. Consequently, the spectral displacements in the SG spectrum reflect the transition from the multicomponent oxide network of the soda–lime glass to a high-purity, hydrated silica gel framework.

For the MS-GP sample, noticeable shifts in these characteristic bands are observed compared to the SG sample. These shifts are primarily attributed to structural rearrangements of the silica network induced by the calcination step used to remove the Pluronic P123 template [44]. Thermal treatment at elevated temperatures promotes changes in Si–O–Si bond angles and local network densification, which are known to result in shifts of vibrational bands toward higher wavenumbers [45,46]. Similar structural rearrangements and variations in Si–O–Si connectivity have been previously reported for silica-based glasses and glass-ceramics subjected to thermal and electrical characterization studies [47]. In this context, the calcination at 550 °C, applied to the MS-GP, increased Si–O–Si bond angles and induced partial condensation of surface silanol groups, explaining the observed spectral displacements.

### 3.4. Zeta Potential Analysis (ξ)

**Figure 7:**
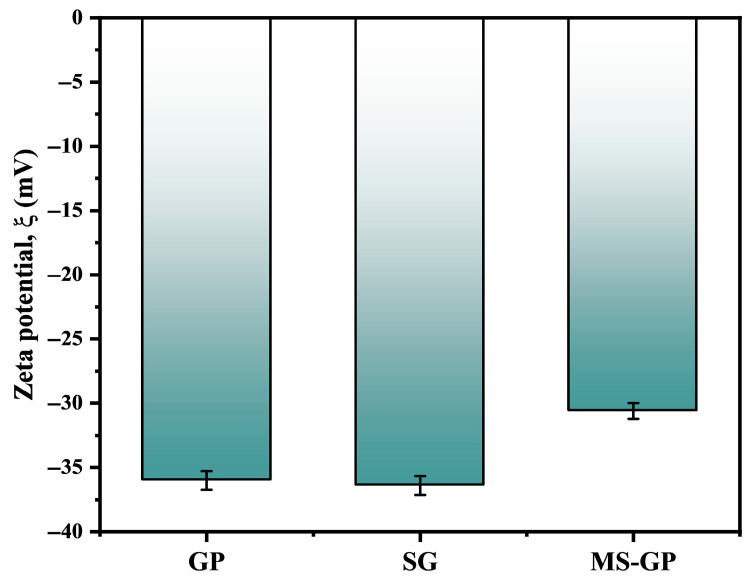
Zeta potential of GP, SG, and MS-GP samples.

The surface charge and colloidal stability of the GP, SG, and MS-GP samples were evaluated via zeta potential (ξ) measurements in phosphate-buffered saline (PBS) at pH 7.4. The results, presented in Figure 7, show that all samples exhibit a negative surface charge, with values of −36.0 mV, −36.4 mV, and −30.6 mV for GP, SG, and MS-GP, respectively. The negative surface charge is attributed to the presence of silanol groups (Si–OH) on the silica surface, which partially deprotonate to form negatively charged silanolate groups (Si–O^−^) under near-neutral or slightly basic conditions [48].

Furthermore, the magnitude of the zeta potential provides important insight into the colloidal stability of particles in aqueous suspensions. In general, absolute zeta potential values greater than approximately 30 mV, either positive or negative, are associated with moderate to good electrostatic stabilization due to interparticle repulsion, which limits aggregation. The values obtained in this study indicate that the waste-derived silica particles exhibit good dispersion stability in aqueous media. This characteristic, combined with their negative surface charge, makes these materials promising candidates for biomedical applications, such as targeted drug delivery systems and cancer therapy, where colloidal stability under physiological conditions is essential [49,50,51].

### 3.5. Nitrogen Sorption Analysis

**Figure 8:**
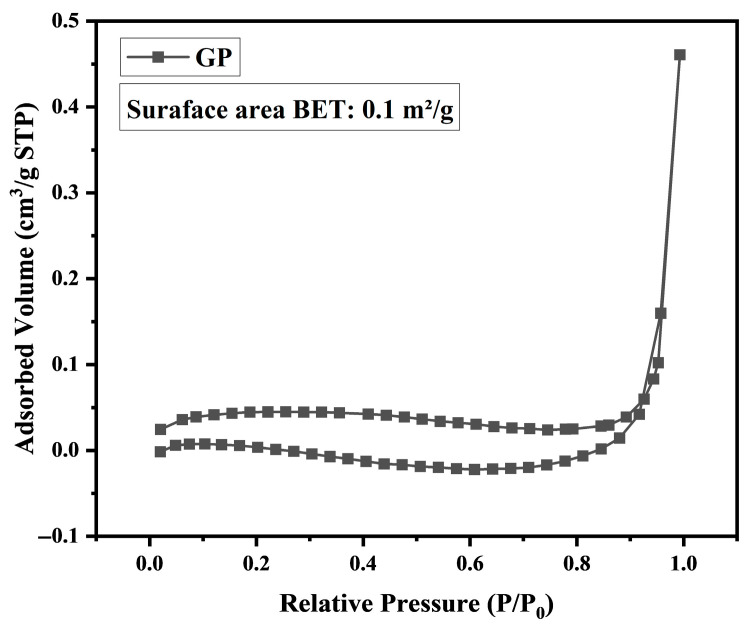
N_2_ sorption isotherms for the GP sample.

The N_2_ adsorption–desorption isotherm of the GP sample is shown in Figure 8. The GP sample exhibits negligible nitrogen uptake, resulting in a very low specific surface area of 0.1 m^2^/g. This behavior is typical of dense, non-porous glass materials and confirms the absence of accessible micro or mesoporosity in the glass powder.

**Figure 9:**
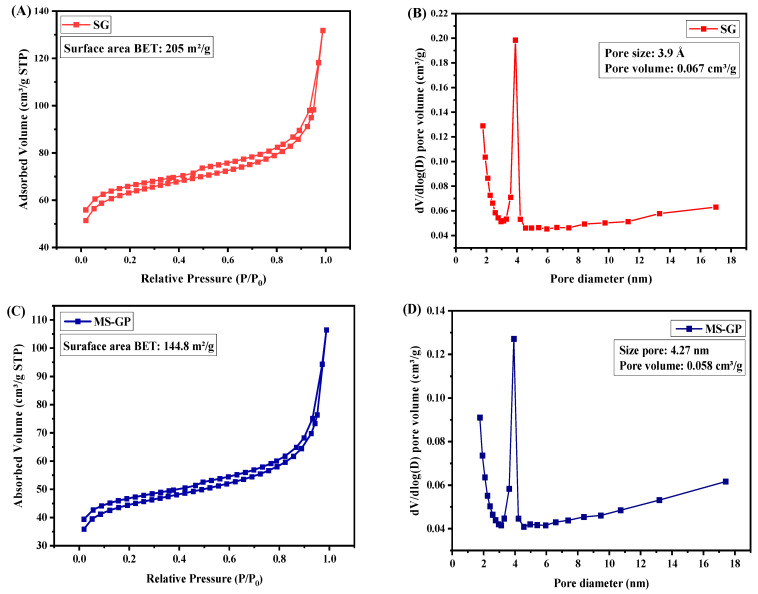
N_2_ sorption isotherms for (**A**) SG and (**C**) MS-GP. Size distribution for (**B**) GS and (**D**) MS-GP.

Figure 9 shows the N_2_ adsorption–desorption isotherms and pore size distributions of the SG and MS-GP samples. Both samples exhibit type II isotherms according to IUPAC classification, which are characteristic of non-porous or macroporous solids and materials with limited mesoporosity. A weak and narrow hysteresis loop is observed at high relative pressures (P/P0 ≳ 0.8), indicating the presence of limited capillary effects. This narrow hysteresis behavior is commonly associated with open, heterogeneous, and weakly connected pore systems and is attributed to textural porosity rather than to a well-developed, ordered mesoporous network. The main textural parameters derived from the adsorption–desorption data are summarized in Table 2.

As shown in Figure 9A,C, the BET surface areas of SG and MS-GP are approximately 205 m^2^/g and 145 m^2^/g, respectively. These values are significantly lower than those typically reported for highly ordered SBA-15 materials synthesized from alkoxide precursors such as TEOS or TMOS, which generally exhibit Type IV isotherms and specific surface areas in the range of 500–1300 m^2^/g [52,53].

The reduced surface area observed for the MS-GP sample can be attributed to the limited solubilization of the silica gel under acidic synthesis conditions, which hindered effective interaction between silica species and the P123 surfactant micelles. Moreover, the calcination step at 550 °C used to remove the organic template can promote further condensation of silanol groups and partial densification of the silica framework, leading to a reduction in accessible surface area [54,55].

The pore size distribution curves of SG and MS-GP samples, presented in Figure 9B,D, possess average pore diameters in the mesoporous range, with the MS-GP sample exhibiting larger pores (Table 2). The gradual increase in pore volume observed at pore diameters above approximately 8 nm suggests the presence of larger mesopore-sized cavities associated with textural porosity or poorly connected pore domains rather than a uniform, well-ordered mesoporous channel system. Such features are commonly associated with pore coalescence, partial pore blocking, or mesopore disconnection, particularly when silica condensation is incomplete or heterogeneous [56]. The presence of larger pores reduces the overall surface area by decreasing the contribution of smaller, high-surface-area pores, which explains the reduced BET values despite measurable pore volumes [57].

Overall, the N_2_ sorption results indicate that while the SG and MS-GP samples exhibit some degree of mesoporosity, the formation of a highly ordered SBA-15-type mesoporous structure is limited. These results are consistent with the weak hysteresis observed in the adsorption–desorption isotherms and with the absence of long-range mesostructural ordering in small-angle XRD, as well as the particle aggregation observed by SEM. The results highlight the critical influence of precursor solubility and synthesis conditions on mesostructure formation when waste-derived silica sources are employed. Several strategies may be considered to improve silica solubility, including enhanced alkaline activation, controlled pH adjustment during dissolution, or the use of alternative dissolution agents.

## 4. Conclusions

In this work, waste soda–lime glass was successfully valorized as a silicon source through alkaline dissolution using NaOH (6 mol·L^−1^), leading to the formation of silica gel with uniformly distributed, spherical particles. Structural characterization confirmed that all samples retained an amorphous silica structure, while FTIR analysis revealed shifts in Si–O–Si vibrational bands after sol–gel processing and calcination, indicating modifications in bond angles and silica network connectivity. N_2_ sorption analysis revealed that the specific surface areas of silica gel and the mesoporous silica samples were comparable. This behavior was attributed to limitations in silica gel solubilization under acidic synthesis conditions, which constrained effective interaction with the surfactant template and hindered the development of a highly ordered mesostructure. Overall, this study demonstrates a proof of concept for the use of waste glass-derived silica gel as a precursor for mesoporous silica synthesis, while explicitly highlighting the structural limitations associated with precursor solubility and synthesis conditions. The results emphasize that careful control of precursor chemistry, solubility, and pH is essential to achieve improved mesostructural ordering when waste-derived silica sources are employed.

## Figures and Tables

**Table 1:** Sample identification and descriptions of the materials prepared in this study.

Sample Code	Description	Structural Characteristic
GP	Glass powder	Amorphous, non-porous
SG	Silica gel derived from glass powder	Amorphous, texturally porous silica
MS-GP	Mesoporous silica synthesized with P123	Disordered mesoporous network

**Table 2:** Textural properties of glass-derived materials obtained from N_2_ adsorption–desorption analysis.

Sample	BET Surface Area (m^2^/g)	Pore Size (Å)	Pore Volume (cm^3^/g)
GP	0.1	—	—
SG	205	3.9	0.067
MS-GP	144.8	4.27	0.058

## Data Availability

The original contributions presented in this study are included in the article. Further inquiries can be directed to the corresponding authors.
